# Reconciling differences in natural tags to infer demographic and genetic connectivity in marine fish populations

**DOI:** 10.1038/s41598-018-28701-6

**Published:** 2018-07-09

**Authors:** Patrick Reis-Santos, Susanne E. Tanner, Maria Ana Aboim, Rita P. Vasconcelos, Jean Laroche, Grégory Charrier, Montse Pérez, Pablo Presa, Bronwyn M. Gillanders, Henrique N. Cabral

**Affiliations:** 1 MARE - Marine and Environmental Sciences Centre, Faculdade de Ciências, Universidade de Lisboa, Campo Grande, 1749-016 Lisboa, Portugal; 2 Southern Seas Ecology Laboratories, School of Biological Sciences, The University of Adelaide, Adelaide, SA 5005 Australia; 3 Departamento de Biologia Animal, Faculdade de Ciências, Universidade de Lisboa, Campo Grande, 1749-016 Lisboa, Portugal; 4 Instituto Português do Mar e da Atmosfera, Rua Alfredo Magalhães Ramalho, 6, 1495-006 Lisboa, Portugal; 5 Université de Bretagne Occidentale Laboratoire des Sciences de l’Environnement Marin (LEMAR, UMR 6539 CNRS/UBO/IRD/Ifremer), Institut Universitaire Européen de la Mer (IUEM), 29280 Plouzané, France; 6 Instituto Español de Oceanografia (IEO), Centro Oceanográfico de Vigo, 36390 Vigo, Spain; 7 University of Vigo, Department of Biochemistry, Genetics and Immunology, 36310 Vigo, Spain

## Abstract

Processes regulating population connectivity are complex, ranging from extrinsic environmental factors to intrinsic individual based features, and are a major force shaping the persistence of fish species and population responses to harvesting and environmental change. Here we developed an integrated assessment of demographic and genetic connectivity of European flounder *Platichthys flesus* in the northeast Atlantic (from the Norwegian to the Portuguese coast) and Baltic Sea. Specifically, we used a Bayesian infinite mixture model to infer the most likely number of natal sources of individuals based on otolith near core chemical composition. Simultaneously, we characterised genetic connectivity via microsatellite DNA markers, and evaluated how the combined use of natural tags informed individual movement and long-term population exchange rates. Individual markers provided different insights on movement, with otolith chemistry delineating Norwegian and Baltic Sea sources, whilst genetic markers showed a latitudinal pattern which distinguished southern peripheral populations along the Iberian coast. Overall, the integrated use of natural tags resulted in outcomes that were not readily anticipated by individual movement or gene flow markers alone. Our ecological and evolutionary approach provided a synergistic view on connectivity, which will be paramount to align biological and management units and safeguard species’ biocomplexity.

## Introduction

Connectivity plays a key role in shaping fish species persistence and responses to harvesting and environmental change^1,2^. The processes driving population homogenization or differentiation are complex and range from extrinsic oceanographic and environmental factors, to intrinsic individual-based or species’ specific features (e.g. larval duration, swimming ability, behaviour, life history strategy)^3^. In tandem, selective pressures and adaptation to gradients of environmental conditions influence population dynamics and connectivity over ecological and evolutionary time scales^4,5^.

Understanding population structure is paramount to conservation and fisheries decision making, particularly for species with large transboundary distribution ranges, or whose life histories include spatially segregated life stages or migratory behaviour. Marine fish populations range from broad and homogeneous to complex and interconnected units^6–8^. Yet, disparities between actual biologically discrete populations and management units are still common^9–11^, and this mismatch severely impacts resource sustainability, and hinders socio-economic and biodiversity goals^12,13^.

A variety of techniques can be used to infer connectivity and population structure, including indirect estimates via abundance-distribution data or life history parameters, or direct quantifications via artificial or natural tags (e.g. phenotypic, genetic, parasitic markers, otolith chemistry)^14^. In particular, otolith chemistry is an exceptional marker of fish movement and key to delineating fish populations and management boundaries^2,15,16^, as otoliths are metabolically inert, grow continuously and their chemical composition is in part influenced by physical and chemical properties of the surrounding environment^17^. Overall, these biogenic carbonate structures are chronological recorders of the environment fish experience throughout their lifetime, but also reflect genetic and physiological influences on element incorporation^18–20^, which partly explains their success in discriminating populations within the more homogeneous marine environment^21^.

The application of otolith chemistry as natural tags for connectivity studies generally relies on first establishing baseline references from source groups (e.g. individuals in spawning or nursery areas). Then individuals of unknown origin collected from mixed populations are retrospectively assigned to characterized source groups via *a priori* statistical classification approaches (e.g. classification, maximum likelihood analysis)^2,15,22^. However, the inherent assumption that all potential source sites are characterized is hardly met, and likely unattainable due to sampling limitations or knowledge gaps in species ecology and distribution. This leads to biased assignments as these methods do not allow for unsampled sources beyond the characterized baseline. Therefore, there is growing recognition that frameworks that incorporate uncertainty and resolve the challenge of unknown sources in mixed populations are key to connectivity assessments^23–25^. In particular, Neubauer *et al*.^26^ developed and validated an unconditional Bayesian mixture model, incorporating the main requirements and assumptions employed in otolith chemistry studies, that allows for unknown (infinite) groups and estimates the most likely number of sources represented in a mixed sample.

Concurrently, the use of genetic markers, particularly allele frequency of microsatellite DNA and single-nucleotide polymorphism assays, have long been based on Bayesian methods and are pivotal in assessments of population structure, connectivity and dispersal in marine fish^27,28^. Overall, dispersal and reproductive movements play a key role in shaping genetic diversity, with many marine fish stocks being amalgamations of multiple geographic components^29^. Genetic heterogeneity implies limits in connectivity and reproductive movements, with spawning site fidelity over individual life time and over many generations increasing the potential for genetic variability among populations^7,30^. Overall, if we lack a clear understanding on genetic structure, life history diversity and the boundaries of biological populations, fisheries management initiatives will fail to protect discrete units or species’ biocomplexity, namely spawning habitats and potential local adaptations^10,29^.

Recently, there has been an increase in the application of multi-marker approaches to address population structure and connectivity in fish^11,31,32^. The rationale is that natural markers are generated by different biological processes and operate over different spatial and temporal resolutions. In particular, otolith chemistry provides demographic information over an individual’s life time whilst genetic markers resolve connectivity and population structure over generational or evolutionary time scales, depending on the rate of variation at given loci^33–35^. These complementary approaches encompass distinct ecological and evolutionary time scales and allow us to unravel individual or stochastic movements as well as to estimate long term population exchange rate and adaptation. In addition, combining them may allow us to overcome the potential limitations of single marker approaches. For example, the lack of spatial variation in otolith chemical composition may not necessarily imply that fish have a common source and may just reflect similar extrinsic and intrinsic factors. In these circumstances, genetic markers may potentially reveal population structuring. Nevertheless, the use of a single framework to truly integrate continuous (i.e. otolith chemistry) and categorical (i.e. genetic markers) data types is still rare^36^, and we should continue to strive to harness the full power of complementary and synergistic interdisciplinary approaches^37,38^.

**Figure 1:**
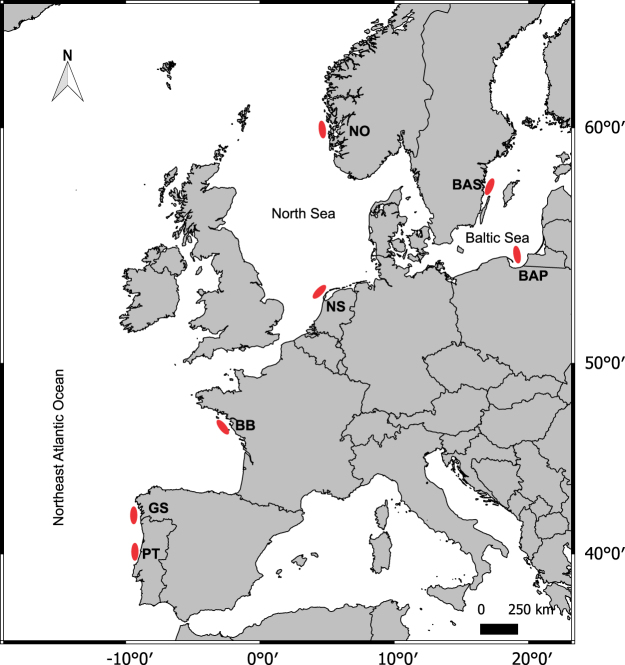
Sampling areas of *Platichthys flesus* in the northeast Atlantic. Sampling areas and abbreviations are: NO – Norwegian coast, BAS – Baltic Swedish coast, BAP – Baltic Polish coast, NS – North Sea, BB – Bay of Biscay, GS – Galician shelf, PT – Portuguese coast. Map was generated using QGIS Version 2.16.2^79^ and made with Natural Earth. Free vector and raster map data @ naturalearthdata.com. Countries vectorial layer version 3.1.0.

Many commercially important fish species have large transboundary distributions throughout the northeast Atlantic, where waning recruitment and decreased spawning stock biomass have led to precautionary or emergency management measures^39^. Yet, for most marine species we lack baseline information on connectivity, relative importance of spawning grounds, or population structure and genetic differentiation on which to base management initiatives^12^. Here we use a Bayesian infinite mixture model to characterize population structure of a flatfish species, European flounder *Platichthys flesus* (L.), throughout the northeast Atlantic (Fig. 1) using the chemical composition of otolith near core regions (representing marine early life stages), whilst simultaneously characterizing the genetic connectivity of these populations. Specifically, we *i*) assessed the spatial separation of early life history stages, estimating the most likely number of source groups (natal origins) to the mixed populations based on otolith chemical composition and using a Bayesian infinite mixture model^26^; *ii*) examined the genetic differentiation of these populations using microsatellite DNA markers; and *iii)* evaluated the combined use of these two marker types with distinct ecological and evolutionary resolutions to support biologically defined management initiatives.

## Results

The numbers of otolith (N_oto_ = 102) and genetic samples (N_gen_ = 318) as well as the number of individuals used in the combined analysis (N_combi_ = 99) per collection location are shown in Table 1.

**Table 1:** Number of individuals of *Platichthys flesus* per sampled location in the northeast Atlantic Ocean and the Baltic Sea used for otolith chemistry (N_oto_), genetic diversity (N_gen_) and combined marker (N_combi_) analyses.

Location	N_oto_	N_gen_	N_combi_
Norwegian coast (NO)	10	28	10
Baltic Swedish coast (BAS)	13	42	13
Baltic Polish coast (BAP)	18	50	18
North Sea (NS)	17	50	17
Bay of Biscay (BB)	13	20	13
Galician shelf (GS)	13	61	12
Portuguese coast (PT)	18	67	16

### Otolith chemistry

**Figure 2:**
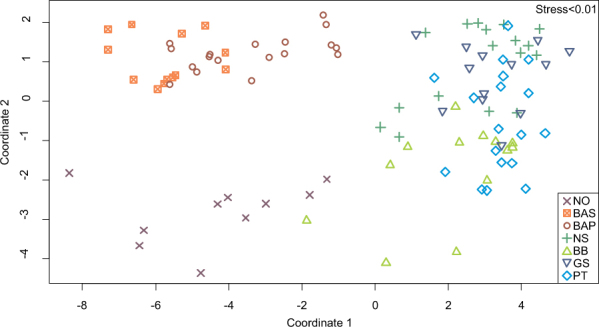
Multidimensional Scaling (MDS) plot based on otolith near core chemical composition of *Platichthys flesus* collected in seven areas along the northeast Atlantic Ocean and the Baltic Sea. Sampling areas and abbreviations are: NO – Norwegian coast, BAS – Baltic Swedish coast, BAP – Baltic Polish coast, NS – North Sea, BB – Bay of Biscay, GS – Galician shelf, PT – Portuguese coast.

Spatial patterns were evident in the MDS based on otolith near core chemical composition, namely individuals collected in the Baltic Sea (Swedish and Polish coast) and the Norwegian coast were clearly segregated from each other and from the remaining samples. Otolith composition in fish from the remaining collection locations covering the North Sea, Bay of Biscay and the Atlantic Iberian Peninsula overlapped in multidimensional space, though with some evidence that individuals from the Bay of Biscay separated more from the remaining locations (Fig. 2).

**Figure 3:**
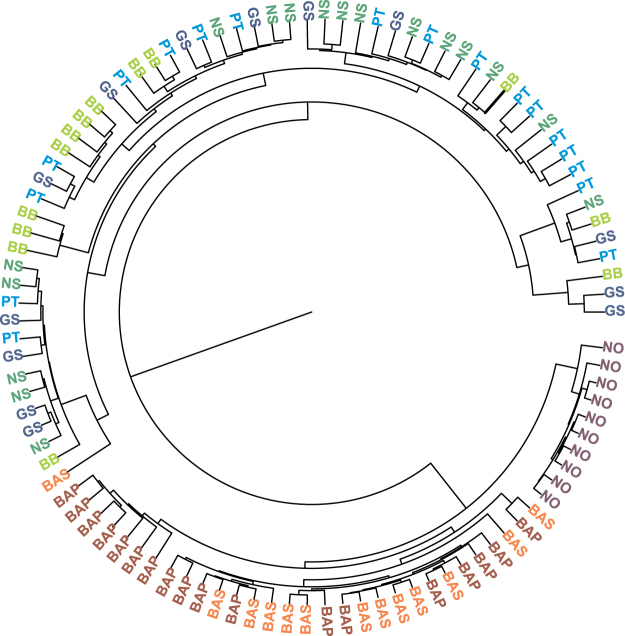
Circular tree of posterior co-assignment probabilities with individuals (abbreviations on tree leaves) grouped into potential sources from the infinite mixture model (*unmixR*) based on the near core chemical composition of *Platichthys flesus*. Most probable number of clusters K = 2. Sampling areas and abbreviations are: NO – Norwegian coast, BAS – Baltic Swedish coast, BAP – Baltic Polish coast, NS – North Sea, BB – Bay of Biscay, GS – Galician shelf, PT – Portuguese coast.

Using the unconditional Bayesian mixture model approach on otolith near core chemical composition, two main clusters were identified (Fig. 3). The first cluster was comprised of individuals collected in the Baltic Sea (Swedish and Polish coasts) and the Norwegian coast, and the second of individuals from the remaining collection locations, though one individual from the Swedish coast was included. Within the first cluster, fish collected in the Norwegian coast waters were grouped together and were indicative of a single source area. For individuals collected from the Baltic Sea, there was some differentiation in the chemical composition of otolith near cores between the Polish and the Swedish coasts, although a few Polish samples were interspersed with Swedish samples. In general, otolith near core chemical composition showed no evidence of structuring from the North Sea to the Atlantic Iberian Peninsula as individuals from these areas were mixed within the second main cluster (Fig. 3).

### Microsatellite markers

Signs of null alleles were detected but only for limited situations across loci and populations. Significant null allele signature is related to heterozygote deficit and therefore to deviations from Hardy–Weinberg equilibrium (HWE). Deviations from HWE were only randomly significant in limited cases (Table 2). Thus, all loci were used in the analyses. Parameters of genetic diversity of *P*. *flesus* including the mean number of alleles per locus, expected heterozygosity (*H*_*e*_) and mean allelic richness (AR) are summarized in Table 2. The within population genetic diversity was similar over the whole data set (0.52 < *H*_e_ < 0.61; 4.78 < AR < 5.59). Pairwise comparisons of genetic differentiation between populations (*F*_ST_ values) were significant with the exception of the Polish coast samples versus North Sea samples and the two most southern locations (Galician shelf and Portuguese coast), with the highest differentiation attained between the Swedish and the Portuguese coasts. (Table 3).

**Table 2:** Measures of genetic diversity for 12 microsatellites of *Platichthys flesus* sampled in seven locations in the northeast Atlantic and the Baltic Sea.

Location		NPlaf_08	NPlaf_27	NPlaf_35	NPlaf_23	NPlaf_15	NPlaf_51	NPlaf_33	NPlaf_25	NPlaf_24	NPlaf_14	NPlaf_30	NPlaf_28	Multilocus
Norwegian coast	N_A_	3	10	4	5	4	6	13	4	5	3	2	9	5.670
(N = 28)	*H*_o_	0.464	0.750	0.607	0.321*	0.429	0.464*	0.750	0.679	0.750	0.250	0.250	0.678	0.533
	*H*_e_	0.447	0.775	0.590	0.465	0.372	0.693	0.823	0.647	0.682	0.312	0.223	0.695	0.561
	*F*_IS_	−0.040	0.034	−0.029	0.314	0.155	0.334	0.090	−0.049	−0.101	0.203	−0.125	0.025	—
	AR	2.991	8.867	3.871	4.571	3.896	5.479	10.841	3.679	4.579	2.970	1.999	7.585	5.110
Baltic Swedish coast	N_A_	4	13	5	4	4	7	11	4	5	3	1	6	5.580
(N = 42)	*H*_o_	0.214	0.816***	0.684**	0.317	0.512	0.685	0.795	0.619	0.625	0.381	—	0.615	0.522
	*H*_e_	0.258	0.757	0.679	0.325	0.476	0.675	0.722	0.535	0.638	0.492	—	0.663	0.518
	*F*_IS_	0.171	−0.079	−0.008	0.024	−0.077	−0.015	−0.102	−0.159	0.021	0.228	—	0.073	—
	AR	3.287	9.627	4.912	3.862	3.978	5.861	8.537	3.976	3.949	2.989	1	5.327	4.780
Baltic Polish coast	N_A_	6	10	6	5	5	6	19	4	8	3	4	9	7.080
(N = 50)	*H*_o_	0.460**	0.673***	0.720	0.580	0.367	0.740	0.920	0.580	0.660	0.200	0.280	0.640	0.568
	*H*_e_	0.521	0.819	0.707	0.534	0.468	0.711	0.872	0.699	0.647	0.246	0.250	0.674	0.595
	*F*_IS_	0.118	0.179	−0.019	−0.088	0.217	−0.041	−0.056	0.172	−0.121	0.188	−0.120	0.051	—
	AR	4.374	8.409	4.753	3.674	4.347	4.912	13.777	3.948	5.950	2.378	2.994	7.193	5.560
North Sea	N_A_	4	10	4	6	5	7	21	6	6	5	4	9	7.250
(N = 50)	*H*_o_	0.340	0.660	0.563	0.540	0.560	0.560	0.755	0.667*	0.638	0.271	0.480	0.739	0.564
	*H*_e_	0.397	0.818	0.649	0.509	0.615	0.645	0.846	0.676	0.649	0.364	0.395	0.778	0.612
	*F*_IS_	0.146	0.196	0.136	−0.016	0.090	0.133	0.108	0.014	0.016	0.259	−0.218	0.051	—
	AR	3.361	8.022	3.396	3.998	4.613	5.217	13.051	4.789	4.841	3.576	3.623	7.684	5.510
Bay of Biscay	N_A_	4	8	5	3	4	5	15	4	6	3	3	7	5.580
(N = 20)	*H*_o_	0.600	0.842	0.700	0.300	0.600	0.600	0.789	0.600	0.684	0.400	0.400	0.421***	0.578
	*H*_e_	0.491	0.832	0.723	0.349	0.481	0.690	0.888	0.643	0.574	0.396	0.343	0.777	0.599
	*F*_IS_	−0.229	−0.012	0.033	0.143	−0.256	0.133	0.133	0.069	−0.197	−0.010	−0.169	0.465	—
	AR	3.949	8.000	4.999	3.000	3.950	4.900	15.000	4.000	6.000	2.950	2.999	7.000	5.560
Galician shelf	N_A_	4	11	5	6	4	6	14	6	13	5	5	8	7.250
(N = 61)	*H*_o_	0.377	0.517***	0.720***	0.589	0.343	0.596	0.775	0.379	0.537	0.298***	0.241***	0.384***	0.480
	*H*_e_	0.415	0.817	0.552	0.603	0.408	0.682	0.798	0.385	0.632	0.399	0.314	0.720	0.561
	*F*_IS_	0.092	0.371	−0.308	0.023	0.161	0.128	0.030	0.015	0.152	0.255	0.233	0.468	—
	AR	3.343	9.146	3.617	4.647	3.787	5.590	10.326	4.422	8.134	3.822	3.575	6.609	5.590
Portuguese coast	N_A_	5	11	5	5	4	6	16	5	8	2	2	8	6.420
(N = 67)		0.552	0.734	0.719	0.567	0.373	0.600	0.778*	0.439	0.454	0.242	0.409	0.642	0.542
	*H*_o_	0.495	0.773	0.724	0.628	0.344	0.673	0.725	0.463	0.436	0.259	0.328	0.733	0.549
	*F*_IS_	−0.116	0.050	0.008	0.098	−0.084	0.109	−0.073	0.052	−0.042	0.065	−0.250	0.126	—
	AR	4.110	8.029	4.506	4.403	3.237	5.391	8.728	4.028	5.438	1.999	2.000	6.410	4.860
All	N_A_	7	19	7	11	5	10	28	7	15	7	12	17	

Number of alleles per locus (N_A_), observed heterozygosity (*H*_o_) with significance of its departure from the Hardy–Weinberg Equilibrium (*p < 0.05; **p < 0.01; ***p < 0.001), unbiased ex*p*ected heterozygosity (*H*_e_), fixation index (*F*_IS_) and mean allelic richness (AR), N = sample size.

**Table 3:** Pairwise comparisons of *F*_*ST*_ values between populations of *Platichthys flesus* collected in the northeast Atlantic and the Baltic Sea (*p < 0.05; ***p < 0.001, NS – not significant).

	NO	BAS	BAP	NS	BB	GS	PT
BAS	0.071***	—					
BAP	0.037***	0.068***	—				
NS	0.042***	0.038***	0.010^NS^	—			
BB	0.029*	0.032***	0.039***	0.020*	—		
GS	0.074***	0.068***	0.067***	0.037***	0.040***	—	
PT	0.082***	0.091***	0.088***	0.059***	0.041***	0.036^NS^	—

Bonferroni correction was used to adjust p-value significance. Sampling areas and abbreviations are: NO – Norwegian coast, BAS – Baltic Swedish coast, BAP – Baltic Polish coast, NS – North Sea, BB – Bay of Biscay, GS – Galician shelf, PT – Portuguese coast.

**Figure 4:**
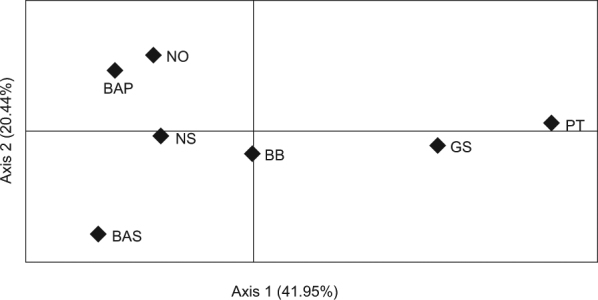
Principal coordinates analysis plot (PCoA) based on 12 microsatellite DNA markers of *Platichthys flesus* along the northeast Atlantic Ocean and the Baltic Sea. Percentage of variation explained by each axis is shown, and sampling areas and abbreviations are: NO – Norwegian coast, BAS – Baltic Swedish coast, BAP – Baltic Polish coast, NS – North Sea, BB – Bay of Biscay, GS – Galician shelf, PT – Portuguese coast.

**Figure 5:**
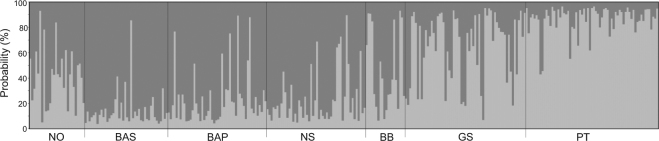
Individual assignment probability based on Bayesian clustering method from STRUCTURE using 12 microsatellite DNA markers. Each bar represents an individual of *Platichthys flesus* with its probability of membership to one of the hypothetical clusters (K = 2). Sampling areas and abbreviations are: NO – Norwegian coast, BAS – Baltic Swedish coast, BAP – Baltic Polish coast, NS – North Sea, BB – Bay of Biscay, GS – Galician shelf, PT – Portuguese coast.

We found a significant isolation by distance (IBD) pattern for *P*. *flesus* in the northeast Atlantic (*r* = 0.7391, *p* < 0.01), and results of PCoA reflected a North-South cline along the first axis, while the second axis clearly separated the two locations within the Baltic Sea (Swedish and Polish coasts). Considering both axes of the PCoA, samples from the Norwegian coast were clustered near the Polish coast and the North Sea (Fig. 4). Furthermore, assignment results of the Bayesian clustering performed in STRUCTURE indicated K = 2 as the most probable number of clusters (Fig. 5). Specifically, individuals from the peripheral and southernmost collection locations (Galician shelf and Portuguese coast) were mostly assigned to the same cluster whilst the remaining locations were included in the other cluster, though high admixture rates were observed in the Norwegian coast and the Bay of Biscay. The latter is a transition area composed of individuals with affinities to both clusters, and although increased admixture in Norway could suggest a third cluster, STRUCTURE results did not support this. Overall, *F*_*ST*_, PCoA and STRUCTURE were indicative of two main regional clusters formed by the southernmost locations (Galician shelf and Portuguese coast) and the more central locations (Bay of Biscay, North Sea and Polish coast), yet they also highlighted increased differentiation of individuals from Norway and the Swedish Coast.

### Chemical and genetic marker integration

**Figure 6:**
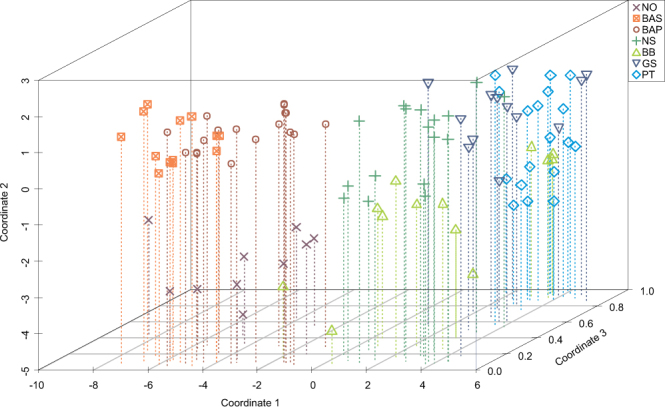
Three-dimensional scatterplot combining the MDS matrix scores from individual otolith data (Coordinates 1 and 2) and the probability of each individual of *Platichthys flesus* belonging to one of the hypothetical clusters in STRUCTURE (Coordinate 3). Sampling areas and abbreviations are: NO – Norwegian coast, BAS – Baltic Swedish coast, BAP – Baltic Polish coast, NS – North Sea, BB – Bay of Biscay, GS – Galician shelf, PT – Portuguese coast.

The combination of results from individual otolith and genetic markers increased the spatial differentiation among locations. The three-dimensional graphical approach highlighted how genetic data contributed to the separation of the central and southern populations of the northeast Atlantic whilst otolith chemical data were more important for disentangling the genetically homogeneous northern locations (Fig. 6). The clear separation between both locations in the Baltic Sea and the Norwegian coast was supported by otolith chemical composition since genetic microsatellite markers were unable to differentiate among these northern locations. Contrastingly, central and southern locations, which otolith chemistry had been unable to separate, were more segregated in the combined approach because of the genetic differentiation of the most southern locations (Galician shelf and Portuguese coast). Overall, integrated marker results suggest the existence of four main groups, namely the Norwegian coast, the Baltic Sea, the North Sea together with the Bay of Biscay, and the Galician shelf together with the Portuguese coast.

## Discussion

The application of different statistical approaches that tackle uncertainty and incomplete baseline data is required to resolve connectivity patterns of commercially exploited marine fish species with large contiguous geographical distributions. Here we applied an infinite Bayesian mixture model to otolith chemical data of *P*. *flesus* collected throughout the northeast Atlantic. Furthermore, we added information from microsatellite DNA markers to otolith chemical data, thus combining two different natural markers with distinct ecological and evolutionary perspectives to increase the likelihood of accurately depicting this species’ population connectivity^11,13,40^.

The infinite mixture model successfully differentiated source groups within our otolith near core chemical data, and in general there was an alignment between these results and the MDS used to visualize data. One of the advantages of the infinite mixture model is that the most probable number of sources is inferred by the algorithm whereas in MDS the identification or separation of groups can be subjective. A clear separation of early life history stages among populations from the Norwegian coast and the Baltic Sea was evident. Individuals from the remaining locations had otolith near core chemical signatures that were not sufficiently differentiated to separate source groups. Identifying the number of sources contributing to a mixed population is a difficult task at best, and in reality likely dependent on the dimensionality of the data and how source groups are segregated in multivariate space^26,41^. Our results represent a first attempt toward this end but in future studies the number of samples to best evaluate population structure of this species using otolith chemistry should be increased. In particular, as in infinite mixture models the number of individual samples needs to increase exponentially with an increasing number of anticipated group signatures^26^.

Regarding otolith chemistry of *P*. *flesus*, no latitudinal gradient was detected from the North Sea to the Iberian Peninsula. Otolith near core chemical composition of individuals collected along the Norwegian coast and the Baltic Sea were clearly differentiated. Although otolith chemistry has been shown to be influenced by physiology and genetics^18,19,21^, environmental factors are still considered the main drivers of variation for certain elements (e.g. Sr, Ba, δ^18^O)^42,43^. In addition to water chemical composition, temperature and salinity may play an important role in the differentiation of the chemical composition of the individuals collected in the two locations within the Baltic Sea and in the northernmost collection site (Norwegian coast). The Baltic Sea is a large brackish water body characterized by strong vertical and horizontal salinity gradients. These gradients are clearly reflected in dissolved inorganic carbon (δ^13^C_DIC_) and water δ^18^O, with more depleted values in low salinity areas^44^ and thus in otoliths^43,45^. Furthermore, otolith chemical studies in the Baltic have shown increased Mn:Ca values due to hypoxia^46,47^. These physico-chemical properties partly contributed to the differentiation of source areas of individuals collected in the Baltic Sea. Similarly, the distinctive otolith chemical composition of individuals from the Norwegian coast is potentially related to lower temperature of this area compared with the remaining collection locations, and varying salinity due to estuarine-like conditions that result from the intricate system of fjords^48^.

Spawning of *P*. *flesus* occurs offshore, and this likely contributed to our inability to differentiate otolith near core compositions of individuals collected from the North Sea to the Iberian Peninsula as the physico-chemical properties of the marine environments in these regions are homogeneous in comparison to near- and inshore environments (e.g. estuaries)^49,50^. Most of the studies attempting to resolve population structure in marine fish species have overlooked the effects of physiology and genetics which can contribute to variations in otolith chemistry and to the success in identifying collection locations in marine environments^19,21^. One key aspect to continue to pursue is identifying other elemental and isotopic markers that may enable the differentiation of locations in marine environments using otolith chemistry. Nevertheless, the possibility of a single source seeding the area from the North Sea to Portuguese waters is highly unlikely and the results of microsatellite DNA markers indicated population structuring in this area. Pelagic larval duration of *P*. *flesus* (25–35 days^51^) allows potential large scale dispersal of early life stages and mixing of contributing sources, yet oceanographic and topographic features may conduce to higher retention rates of early life stages (e.g. Skagerrak Strait, Iberian upwelling system). Overall, connectivity in marine fish cannot be readily predicted from early life history traits and dispersal potential as multiple biophysical processes are involved in shaping connectivity patterns^52^.

Complementary to the otolith chemical markers, microsatellite DNA markers highlighted a genetic split between the Bay of Biscay and the Iberian Peninsula, and in general genetic differentiation followed a latitudinal gradient. Calvès, *et al*.^53^ already showed genetic differentiation between *P*. *flesus* populations from the Mondego estuary (Portugal) and estuaries along the English Channel. In fact, the Portuguese coast is acknowledged as the southern limit of distribution of *P*. *flesus*^54^, and there is likely a combination of adaptive processes to increased temperature and reduced gene flow taking place here due to its peripheral geographic location^55^. Regarding the Norwegian coast, though otolith chemistry suggests this was a well separated source group, additional genetic markers and samples are necessary to substantiate potential genetic divergence of this population. A further spatially saturated sampling design will aid in disentangling the location of the genetic split towards the Iberian populations, and could simultaneously contribute to better understanding the genetic structure of *P*. *flesus* populations at its northern distribution limit. On the other hand, it is well documented that *P*. *flesus* has distinct pelagic and demersal spawning behaviours in the Baltic Sea, and this likely explains the genetic differentiation observed between the two geographically close populations^56,57^.

The combined analysis of otolith chemical and genetic markers on the same individuals increases the likelihood of correctly characterizing population connectivity, as they reflect ecological and evolutionary processes^16,38,58^. An integrated approach should provide more robust conclusions regarding population identity, and this was manifest in our results, as markers provided complementary perspectives on *P*. *flesus* population structure. Both markers showed differentiation among northern and Baltic populations. However, otolith chemistry was more successful in identifying different source groups in these northern areas, whilst only microsatellite DNA markers detected population structuring towards the southern limit of *P*. *flesus* distribution. Considering the different nature of demographic and genetic markers, reconciling contrasting connectivity patterns can be highly informative^16,32,59^, whilst disregarding information from one of them can lead to misconceptions in fish population connectivity. Therefore, synthesizing data from a suite of markers and compiling congruent information at different spatio-temporal scales is the best practice to provide robust information to define and support actions and decisions in fisheries and conservation^10,12,13^.

Despite the increased recognition of the importance of conducting multidisciplinary population assessments based on the consensus and complementarity of multiple markers^37^, we have yet to establish a common statistical approach that can adequately resolve the limitations associated with the different nature of the data obtained from distinct markers. Specifically, one of the main hurdles to overcome is combining the categorical data from genetic markers with the continuous data from most other markers (e.g. otolith chemistry, morphometrics)^13^. On the other hand, Bayesian approaches dealing with probabilities are able to define realistic models that incorporate uncertainty at various levels^26,36,60^, and handle scenarios with unknown sources to a mixed population^26^, as seen here with the infinite mixture model. To date, studies have combined the information resulting from independent analysis of different datasets^31^, synthesized multiple marker information in quantitative or qualitative indices^32^, and developed approaches that allow integration in specific contexts or add information in *a priori* classification-based methods^11^. To our knowledge, only one study has taken full advantage of the available information by effectively integrating otolith chemistry and genetic markers to determine the proportion of groups of origin in a mixed population of Atlantic cod^36^. Adapting Bayesian infinite mixture models to adequately accommodate different data types will contribute towards integration of multiple marker data, and represent a breakthrough in ascertaining the biological boundaries among populations or the sources contributing to mixed populations.

Characterizing the biocomplexity of fish species that span large spatial ranges is critical to avoid mismatches between management and biological units. Here, we showed how the complementary use of two markers with distinct ecological and evolutionary resolutions can enhance our perspective of population connectivity. Results highlighted how single marker approaches may lead to the misinterpretation of a species’ population structure. Overall, disregarding demographic and genetic connectivity in management decisions will have significant ecological and economic consequences, particularly in the current context of general over exploitation of fisheries resources.

## Materials and Methods

### Sample collection and preparation

European flounder *Platichthys flesus* (L.) has a complex life cycle with adults spawning at sea whilst juveniles use nearshore to inshore environments with large-scale movements between the different habitats^15^. A total of 318 individuals of *P*. *flesus* [mean (and standard deviation) of fish total length: 292 (56) mm] were obtained between 2012 and 2014 directly from commercial fishers and research surveys at locations throughout the northeast Atlantic Ocean, including the Baltic Sea (Fig. 1). Collection locations covered the species distribution range and included the Norwegian coast, Swedish coast, Polish coast, North Sea, Bay of Biscay, Galician shelf, and the Portuguese coast, where the species southern limit of distribution occurs^54^. Individual fin tissue clips (ca. 1 cm^2^) were removed and stored in 100% ethanol for genetic analysis (N of individuals analysed for genetic markers = 318, Table 1). Sagittal otoliths were extracted, washed and cleaned of adhering tissues and stored dry in microcentrifuge tubes until further processing. Whole otoliths were immersed in ultrapure water and aged using a binocular stereoscope to ensure all fish used for otolith chemical analysis were spawned in the same year and limit the potential effect of temporal variation on otolith chemical composition^61,62^. Only otoliths of individuals matching the 2009 cohort/birth year (ages 3 to 5) were selected (N of individuals analysed for otolith markers = 102, Table 1). No live animals were used in this study and no specific permissions were needed for the sampling as the species is commercially harvested (not endangered nor protected) and specimens were caught in areas where fishing is allowed.

### Otolith chemistry analysis

Otolith preparation and chemical analysis followed Tanner, *et al*.^50^. Briefly, for elemental analysis eye side (right) otoliths were ground to the midplane using lapping paper (30 µm and 3 µm) until the core was exposed. In a class-100 clean room, otoliths were immersed in ultrapure water, sonicated for 3 min, triple rinsed with ultrapure water, and then mounted on new glass slides in random sets of 30 using double sided tape. All glass and plasticware used for otolith processing were previously decontaminated in a 10% nitric acid (HNO_3_) wash for 24 h, rinsed with ultrapure water and dried in a laminar flow positive pressure fume hood.

Otolith elemental ratios were quantified by measuring ^25^Mg, ^48^Ca, ^55^Mn, ^88^Sr, and ^138^Ba on a Thermo Finnigan Element2 single collector inductively coupled plasma mass spectrometer (ICP-MS) coupled to a New Wave 193 nm excimer laser ablation system. 450 µm long rasters with a 30 µm beam diameter were ablated in the near core region, corresponding to the marine larval life history stage. Ablated material was transported via a He gas stream to the dual-inlet quartz spray chamber and mixed with a 2% HNO_3_ aerosol from a self-aspirating PFA 20 μl min^−1^ nebulizer. Resulting analyte was transported to the ICP-MS via an Ar carrier gas. Instrumental blanks (2% HNO_3_) were run at the beginning and end of each set of 10 otoliths. Blank correction of all measured raw values was done by calculating a blank value for each sample by linear interpolation of the measured blanks. A dissolved otolith certified reference material (CRM)^63^ at Ca concentration of 40 μg g^−1^ was used to correct for instrument mass bias. Instrument precision was assessed by running another CRM^64^, dissolved and diluted to a Ca concentration of 40 μg g^−1^. External precision (relative standard deviation) for this CRM (n = 46) was as follows: Mg:Ca = 0.59%, Mn:Ca = 14.30%, Sr:Ca = 0.51%, and Ba:Ca = 1.29%. All otolith elemental concentration data were converted to element:Ca ratios.

For stable isotope analysis of δ^13^C and δ^18^O, blind side (left) otoliths were ground to the midplane using lapping paper (30 µm and 3 µm) until the core region began to be exposed. A computer-controlled micromill (ESI New Wave Research Micromill) was used to extract otolith material from the near core area using the same sized rasters as for elemental analysis (drilling depth 100 µm) until *c*. 50 µg of otolith material was obtained taking into account the circular structure of otoliths. Thus, otolith material for isotopic analysis represents the same time period as that targeted in the right otolith for elemental analysis. Samples were analyzed on a Thermo Scientific MAT 253 isotope ratio mass spectrometer (IRMS) with dual inlet coupled to a Kiel IV device for automated sample preparation following standard methods^65^. Isotopic values were reported relative to Vienna Pee Dee belemnite (VPDB) and expressed in standard δ notation. Long term analytical uncertainties for δ^13^C and δ^18^O based on repeat measurements of NBS-19 certified reference material are 0.04‰ and 0.06‰, respectively.

### Otolith chemistry data analysis

As there is no prior information regarding potential spawning grounds and larval distributions for *P*. *flesus* throughout the northeast Atlantic, only unconstrained statistical methods were used when assessing near core chemical composition. When sources in mixed samples are uncertain, identifying the origin of individuals becomes a difficult statistical problem as any classification will be biased *a priori* by the exclusion of potential sources^26^. We used multidimensional scaling (MDS) analysis to visualize data in reduced space and examine the occurrence of potential groupings, using *Vegan* package in R. Second, we adapted the unconditional Bayesian mixture model approach (*PopR* package, https://github.com/Philipp-Neubauer/PopR) from Neubauer, *et al*.^26^. This approach implements a Dirichlet process mixture that allows unknown (infinite) sources and estimates the most likely number of sources represented in a mixed sample. However, the core functions of *PopR* are implemented in Julia, which brings gains in computation time but opens up a number of issues. In particular, Julia is a recent programming language and is still being actively developed rendering its functionality and synchronization with R unstable. Thus, to address these issues, the *unmixR* package was developed translating and updating all of *PopR*’s Julia components to C++, making extensive use of the *Rcpp* and *RcppArmadillo* packages. This removed the need for an external interpreter and the creation of intermediate files (*unmixR* package, https://github.com/zeloff/unmixR). No changes were made to the original *PopR* algorithms.

Preliminary analysis of the otolith chemical data indicated that best results were obtained by using both elemental and isotopic variables therefore the full set of variables was used for subsequent analyses. All data treatment and statistical analyses were made in R^66^. Simulation parameters for the *unmixR* analysis were 2000 iterations and thinning was set to 1.

### Microsatellite markers and genotyping

Total genomic DNA was extracted from fin clips using a standard phenol-chloroform extraction protocol^67^. Twelve microsatellites (Nplaf8, Nplaf14, Nplaf15, Nplaf23, Nplaf24, Nplaf25, Nplaf27, Nplaf28, Nplaf30, Npalf33, Npal35, Nplaf51) were grouped into 3 multiplex PCR and genotyping groups^68^. PCR reactions were run with the Type-it Microsatellite PCR Kit (Qiagen TM) containing 5 µl of Master Mix, 1 µl Q-solution, 2 µl RNase-free water, 1 µl of primer mix and 1 µl genomic DNA (*c*. 50 ng). Amplification conditions were: 95 °C for 10 min followed by 40 cycles of 95 °C for 30 s, 55 °C for 30 s and 72 °C for 1 min and an extension step of 10 min at 72 °C^68^.

Samples were genotyped in an ABI PRISM 310 Genetic Analyzer and fragments were sized with GeneScan-500 LIZ Size Standard using GeneMapper 3.7 (Applied Biosystems), and blindly scored by at least two readers to minimize genotyping errors.

### Microsatellite markers data analysis

Microsatellite loci were first tested for the presence of null alleles and genotyping errors like null alleles, stuttering and large allele dropout using MICRO-CHECKER 2.2.3^69^. Genetic diversity was measured as the mean allelic richness (AR) calculated and corrected for sample size by rarefaction using HP-Rare^70^; observed heterozygosity (*H*_o_), unbiased expected heterozygosity (*H*_e_)^71^ both per loci and multilocus, mean number of alleles across loci (N_A_) using ARLEQUIN 3.11^72^; and fixation index within samples (*F*_IS_), inferred using GENETIX 4.05.2^73^. Deviations from HWE and linkage disequilibrium were tested using ARLEQUIN per locus and Genepop 3.1^74^ for all loci, using 100 000 Markov chain and 1000 dememorization steps. ARLEQUIN software was also used to calculate population differentiation through pairwise comparisons of *F*_ST_ (fixation index between samples and 100 000 steps for Exact tests) and p-value significance was assumed using the Bonferroni correction for multiple comparison tests.

Isolation by distance (IBD) was tested by comparing pairwise *F*_*ST*_ values against the shortest waterway distance between collection locations of *P*. *flesus* using a Mantel-test implemented in the *Vegan* package in R. Statistical significance was assessed with 1000 permutations of the data.

Patterns of differentiation were visualized by principal coordinates analysis (PCoA), a multivariate technique that allows major patterns within a multivariate dataset of multiple loci and samples to be discerned. This analysis was computed using GenAlEx 6.5^75^.

A Bayesian clustering method (STRUCTURE 2.3.4)^76^ was used to infer possible genetic clusters, with parameters of 1000000 Monte Carlo Markov Chain interactions following a burn-in period of 1000000 for K = 1–7, as number of tested clusters, and replicated 20 times. Evanno’s index AK^77^ was evaluated using STRUCTURE HARVESTER 0.6.93^78^.

### Integrating genetic and chemical markers

Due to the differing nature of otolith chemistry data and genetic data, a straightforward integration of the datasets is challenging^11,36^. The potential benefits of integrating the two markers were evaluated graphically with a three-dimensional scatterplot combining the probability of each individual belonging to one of the identified genetic clusters and the MDS matrix scores from individual otolith data. Thus, the data used for each individual originated from the results obtained using the full data sets for each marker. One advantage of this integrated graphical approach relies on the combination of results from continuous and categorical data without requiring re-analysis of the data.

### Data availability

The datasets generated and/or analysed during the current study are available from the corresponding author on reasonable request.
